# Supplementary material for: Quality of care in sterilization services at the public health facilities in India: A multilevel analysis
Source: PLoS One. 2020 Nov 2;15(11):e0241499. doi: 10.1371/journal.pone.0241499 (PMC7605679; doi:10.1371/journal.pone.0241499)
Supplement: S1 Table — (DOCX) [file pone.0241499.s001.docx]

**S1 Table 1. Variables used to create the quality of sterilization care index and percentage of women received each of the quality of care item during sterilization, NFHS 2015–16.**

| **List of variables** | **Recoding** | **% of women received (N=145301)** |
| --- | --- | --- |
| Told sterilization would mean no more children | 1 ‘Yes’ 0 ‘No’ | 79.3 |
| Told about side effects of sterilization | 1 ‘Yes’ 0 ‘No’ | 86.3 |
| Told about how to deal side effects of sterilization | 1 ‘Yes’ 0 ‘No’ | 84.8 |
| Told about other family planning methods | 1 ‘Yes’ 0 ‘No’ | 87.3 |
| Rated care received as ‘Good’ or ‘All right’ | 1 ‘Very good’ or ‘All right’ 0 ‘No so good’ or 0 ‘Bad’ | 95.5 |
