# Supplementary material for: Quality of care in sterilization services at the public health facilities in India: A multilevel analysis
Source: PLoS One. 2020 Nov 2;15(11):e0241499. doi: 10.1371/journal.pone.0241499 (PMC7605679; doi:10.1371/journal.pone.0241499)
Supplement: S2 Table — (DOCX) [file pone.0241499.s002.docx]

**S2 Table 2.Variables used to create ‘structure’ index and percentage of facilities not having each of the items on infrastructure and readiness by the type of facilities, DLHS facility survey 2012–13.**

| **List of variables** | **District hospitals** | **Community health centers** | **Pooled** |
| --- | --- | --- | --- |
|  | N= 1540 | N= 4810 | N= 6350 |
|  | % | % | % |
| Availability of 24*7 running water supply | 3.18 | 7.90 | 6.76 |
| Availability of regular power supply | 4.48 | 42.16 | 33.02 |
| Availability of generator/invertor | 2.86 | 9.63 | 7.98 |
| Availability of proper sewage facility | 8.44 | 10.64 | 10.11 |
| Availability of functional ECG machine | 21.10 | 65.53 | 54.76 |
| Availability of functional X-ray machine | 12.08 | 56.05 | 45.39 |
| Availability of functional ultrasound machine | 34.09 | 86.86 | 74.06 |
| Availability of functional operation theatre | 15.13 | 19.79 | 18.66 |
| Availability of functional labor room | 14.16 | 4.14 | 6.57 |
| Availability of functional pharmacy | 1.43 | 4.80 | 3.98 |
| Availability of functional blood bank | 51.10 | 94.37 | 83.87 |
| Availability of functional blood storage facility | 46.75 | 86.61 | 76.94 |
| Availability of functional laboratory services | 3.44 | 7.03 | 6.16 |
| Availability of functional ambulance | 14.29 | 28.13 | 24.77 |
| Availability of functional stock registers | 5.97 | 1.41 | 2.52 |
| Availability of functional OPD registers | 1.49 | 0.98 | 1.10 |
| Availability of delivery registers | 5.97 | 4.20 | 4.63 |
| Availability of IPD registers | 1.88 | 3.26 | 2.93 |
| Facility wise data uploaded on HMIS portal | 7.73 | 5.78 | 6.25 |
| Availability of clean OPD | 1.88 | 3.53 | 3.13 |
| Availability of clean rooms | 2.66 | 4.91 | 4.36 |
| Availability of clean wards | 5.19 | 7.82 | 7.18 |
| Availability of clean premises | 8.05 | 13.41 | 12.11 |
| Availability of primary health nurse | 72.47 | 79.71 | 77.95 |
| Availability of ANM | 24.87 | 23.18 | 23.59 |
| Availability of staff nurse | 7.47 | 9.69 | 9.15 |
| Availability of pharmacist/Compounder | 5.97 | 10.31 | 9.26 |
| Availability of general surgeon | 32.60 | 80.89 | 69.18 |
| Availability of gynecologist | 23.77 | 69.04 | 58.06 |
| Availability of anesthetist | 30.26 | 76.51 | 65.29 |
| Availability of physician | 19.55 | 78.69 | 64.35 |
| Availability of pediatrician | 24.29 | 80.94 | 67.20 |
