# Supplementary material for: Quality of care in sterilization services at the public health facilities in India: A multilevel analysis
Source: PLoS One. 2020 Nov 2;15(11):e0241499. doi: 10.1371/journal.pone.0241499 (PMC7605679; doi:10.1371/journal.pone.0241499)
Supplement: S3 Table — (DOCX) [file pone.0241499.s003.docx]

**S3 Table 3. Background characteristics of the clients.**

| **List of variables** | **Recoding** |
| --- | --- |
| Residence | 1 ‘Urban’ 2 ‘Rural’ |
| Religion | 1 ‘Hindu’ 2 ‘Muslim’ 3 ‘Christian’ 4 ‘Others’ |
| Social groups^1^ | 1 ‘Scheduled caste’ 2 ‘Scheduled tribe’ 3 ‘Other backward classes’ 4 ‘Others’ |
| Wealth index | 1 ‘Poorest’ 2 ‘Poorer’ 3 ‘Middle’ 4 ‘Richer’ 5 ‘Richest’ |
| Education status | 0 ‘No education’ 1 ‘Primary’ 2 ‘Secondary’ 3 ‘Higher’ |
| Region | 1 ‘North’ 2 ‘Central’ 3 ‘East’ 4 ‘Northeast’ 5 ‘West’ 6 ‘South’ |
| Place of sterilization | 1 ‘GH/DH/MH’ 2 ‘CHC’ 3 ‘PHC/Sub-center’ 4 ‘Camp/Mobile clinic/Others’ |

^1 Scheduled caste, scheduled tribes, and other backward classes are marginalized groups in India designated by the government and recognized by the Constitution of India^
