# Supplementary figures and images for: Quality of care in sterilization services at the public health facilities in India: A multilevel analysis
Source: PLoS One. 2020 Nov 2;15(11):e0241499. doi: 10.1371/journal.pone.0241499 (PMC7605679; doi:10.1371/journal.pone.0241499)

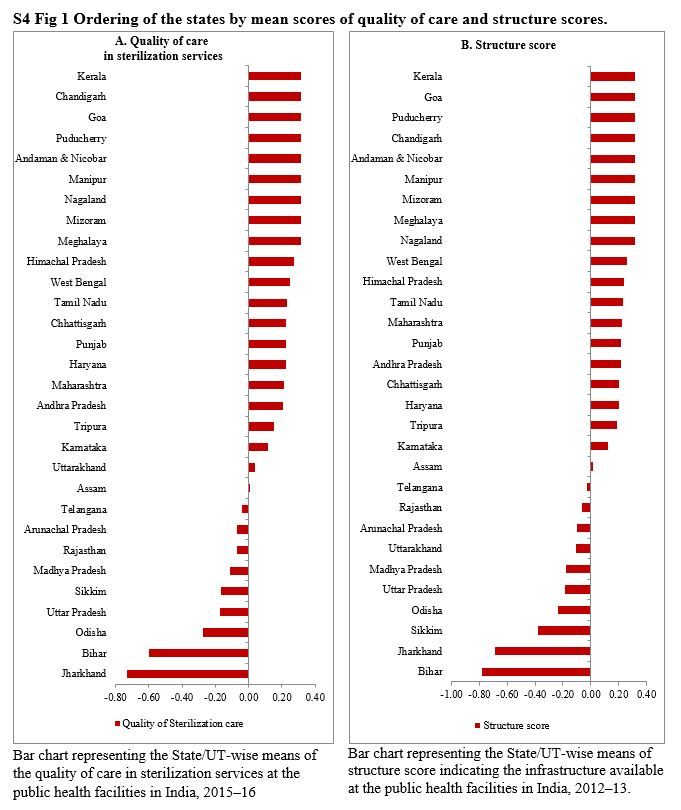

Supplement: S1 Fig — (TIF) [file pone.0241499.s004.tif]
