# Supplementary material for: Quality of care in sterilization services at the public health facilities in India: A multilevel analysis
Source: PLoS One. 2020 Nov 2;15(11):e0241499. doi: 10.1371/journal.pone.0241499 (PMC7605679; doi:10.1371/journal.pone.0241499)
Supplement: Attachment — Submitted filename: 3 Response_to_Reviewers.docx [file pone.0241499.s005.docx]

Dear Dr. Navaneetham,

Thank you for giving us the opportunity to revise the manuscript. We have addressed all the comments made by the reviewers and updated the manuscript according to the style requirements of the journal. Please find the point-by-point replies to the reviewers’ comments, appended below.

Thank you for your consideration.

Sincerely yours

Arupendra Mozumdar

Correspondence Author

Journal Requirements:

When submitting your revision, we need you to address these additional requirements.

 1. Please ensure that your manuscript meets PLOS ONE's style requirements, including those for file naming. The PLOS ONE style templates can be found at

<https://journals.plos.org/plosone/s/file?id=wjVg/PLOSOne_formatting_sample_main_body.pdf> and

<https://journals.plos.org/plosone/s/file?id=ba62/PLOSOne_formatting_sample_title_authors_affiliations.pdf>

Answer: Thank you for noting this. We have updated the manuscript according to the Journal guidelines.

2. Please provide additional details regarding participant consent. In the ethics statement in the Methods and online submission information, please ensure that you have specified what type you obtained (for instance, written or verbal, and if verbal, how it was documented and witnessed). If your study included minors, state whether you obtained consent from parents or guardians. If the need for consent was waived by the ethics committee, please include this information.

Answer: The NFHS 2015–16 and DLHS 2012–13 received ethical clearance from the Ethical Review Board of the International Institute for Population Science, which conducted these surveys. The analysis presented in this study is based on a secondary analysis of existing survey data, available in the public domain without any identifying information. The survey personnel obtained informed consent from each respondent before the interview and made their best effort to maintain privacy.

3. We note that you have indicated that data from this study are available upon request. PLOS only allows data to be available upon request if there are legal or ethical restrictions on sharing data publicly. For information on unacceptable data access restrictions, please see <http://journals.plos.org/plosone/s/data-availability#loc-unacceptable-data-access-restrictions>.

Answer: Data of NFHS are available in the public domain without any identifiers in the following website—https://dhsprogram.com/data/available-datasets.cfm.

Data of DLHS are available in the public domain in the following website—http://rchiips.org/DLHS-4.html.

In your revised cover letter, please address the following prompts:

 a) If there are ethical or legal restrictions on sharing a de-identified data set, please explain them in detail (e.g., data contain potentially identifying or sensitive patient information) and who has imposed them (e.g., an ethics committee). Please also provide contact information for a data access committee, ethics committee, or other institutional body to which data requests may be sent.

 b) If there are no restrictions, please upload the minimal anonymized data set necessary to replicate your study findings as either Supporting Information files or to a stable, public repository and provide us with the relevant URLs, DOIs, or accession numbers. Please see <http://www.bmj.com/content/340/bmj.c181.long> for guidelines on how to de-identify and prepare clinical data for publication. For a list of acceptable repositories, please see <http://journals.plos.org/plosone/s/data-availability#loc-recommended-repositories>.

Answer: Data are available in public domain.

4. Please ensure that you refer to Figure 1 in your text as, if accepted, production will need this reference to link the reader to the figure.

Answer: Thank you for noting this. As you suggested, we have cited the Figure.

5. We note that Figures 2 and 3 in your submission contain map images which may be copyrighted. All PLOS content is published under the Creative Commons Attribution License (CC BY 4.0), which means that the manuscript, images, and Supporting Information files will be freely available online, and any third party is permitted to access, download, copy, distribute, and use these materials in any way, even commercially, with proper attribution. For these reasons, we cannot publish previously copyrighted maps or satellite images created using proprietary data, such as Google software (Google Maps, Street View, and Earth). For more information, see our copyright guidelines: <http://journals.plos.org/plosone/s/licenses-and-copyright>.

Answer: The authors created those original maps in Figures 2 and 3 from NFHS-4 and DLHS-4 data, respectively. Therefore, authors currently hold copyright.

The shapefiles used to create Fig 2, and Fig 3 were downloaded from the DHS program spatial data repository, which can be downloaded without any restriction along with the NFHS data. For more details visit DHS website to obtain the GPS datasets.

https://dhsprogram.com/data/available-datasets.cfm

One of the authors (VJ) has modified the boundaries of Jammu & Kashmir region in the shapefile using GIS mapping software so that the map displays in accordance to the official map recognized by the government of India. The author has the modified shapefile.

We require you to either (1) present written permission from the copyright holder to publish these figures specifically under the CC BY 4.0 license, or (2) remove the figures from your submission:

 5. 1.    You may seek permission from the original copyright holder of Figures 2 and 3 to publish the content specifically under the CC BY 4.0 license.

 We recommend that you contact the original copyright holder with the Content Permission Form (<http://journals.plos.org/plosone/s/file?id=7c09/content-permission-form.pdf>) and the following text:

“I request permission for the open-access journal PLOS ONE to publish XXX under the Creative Commons Attribution License (CCAL) CC BY 4.0 (<http://creativecommons.org/licenses/by/4.0/>). Please be aware that this license allows unrestricted use and distribution, even commercially, by third parties. Please reply and provide explicit written permission to publish XXX under a CC BY license and complete the attached form.”

 Please upload the completed Content Permission Form or other proof of granted permissions as an "Other" file with your submission.

 In the figure caption of the copyrighted figure, please include the following text: “Reprinted from [ref] under a CC BY license, with permission from [name of publisher], original copyright [original copyright year].”

 5.2.    If you are unable to obtain permission from the original copyright holder to publish these figures under the CC BY 4.0 license or if the copyright holder’s requirements are incompatible with the CC BY 4.0 license, please either i) remove the figure or ii) supply a replacement figure that complies with the CC BY 4.0 license. Please check copyright information on all replacement figures and update the figure caption with source information. If applicable, please specify in the figure caption text when a figure is similar but not identical to the original image and is therefore for illustrative purposes only.

The following resources for replacing copyrighted map figures may be helpful:

 USGS National Map Viewer (public domain): <http://viewer.nationalmap.gov/viewer/>

The Gateway to Astronaut Photography of Earth (public domain): <http://eol.jsc.nasa.gov/sseop/clickmap/>

Maps at the CIA (public domain): <https://www.cia.gov/library/publications/the-world-factbook/index.html> and <https://www.cia.gov/library/publications/cia-maps-publications/index.html>

NASA Earth Observatory (public domain): <http://earthobservatory.nasa.gov/>

Landsat: <http://landsat.visibleearth.nasa.gov/>

USGS EROS (Earth Resources Observatory and Science (EROS) Center) (public domain): [http://eros.usgs.gov/#](http://eros.usgs.gov/)

Natural Earth (public domain): <http://www.naturalearthdata.com/>

[Note: HTML markup is below. Please do not edit.]

Reviewers' comments:

Reviewer's Responses to Questions

**Comments to the Author**
1. Is the manuscript technically sound, and do the data support the conclusions?

The manuscript must describe a technically sound piece of scientific research with data that supports the conclusions. Experiments must have been conducted rigorously, with appropriate controls, replication, and sample sizes. The conclusions must be drawn appropriately based on the data presented.

Reviewer #1: Partly

Reviewer #2: Yes

2. Has the statistical analysis been performed appropriately and rigorously?

Reviewer #1: Yes

Reviewer #2: Yes

3. Have the authors made all data underlying the findings in their manuscript fully available?

The [PLOS Data policy](https://nam02.safelinks.protection.outlook.com/?url=http%3A%2F%2Fwww.plosone.org%2Fstatic%2Fpolicies.action%23sharing&data=02%7C01%7Camozumdar%40popcouncil.org%7Cd216b84cb1ac48fab92a08d83ef1cef5%7Cda295b63c8454fa2990b1ab42d54f2ca%7C1%7C0%7C637328556557106090&sdata=iKD6xdPsIjtPbjrpUy832BlToC%2Bx0ibh8WyEuP64aBM%3D&reserved=0) requires authors to make all data underlying the findings described in their manuscript fully available without restriction, with rare exception (please refer to the Data Availability Statement in the manuscript PDF file). The data should be provided as part of the manuscript or its supporting information, or deposited to a public repository. For example, in addition to summary statistics, the data points behind means, medians and variance measures should be available. If there are restrictions on publicly sharing data—e.g. participant privacy or use of data from a third party—those must be specified.

Reviewer #1: Yes

Reviewer #2: No

4. Is the manuscript presented in an intelligible fashion and written in standard English?

PLOS ONE does not copyedit accepted manuscripts, so the language in submitted articles must be clear, correct, and unambiguous. Any typographical or grammatical errors should be corrected at revision, so please note any specific errors here.

Reviewer #1: Yes

Reviewer #2: Yes

5. Review Comments to the Author

Please use the space provided to explain your answers to the questions above. You may also include additional comments for the author, including concerns about dual publication, research ethics, or publication ethics. (Please upload your review as an attachment if it exceeds 20,000 characters)

Reviewer #1: Dear authors,
Overall, this was a good paper and nice to see more research linking facility and household survey data. I have some comments for further clarifications and some suggestions for your analysis. I hope my suggestions will further improve your paper.
Thank you.

1. Quality of care index outcome – The variables listed in Appendix 1 are mainly related to counseling or what was told to the client by the provider. Are there some other components of QoC that can also be added? What about ensured privacy (private room during consultation), or following medical guidelines (washed hands, sanitized equipment) that may be observed during the survey.

Answer: Authors agree with the reviewer that other QoC indicators such as privacy or following the standard of protocols are important. However, NFHS does not provide those data. This study used only those QoC indicators which are available in the NFHS data, and those five variables were included in the analyses.

2. How were the two datasets linked? The NFHS data have displacement procedures to protect the privacy of individuals. The facility survey is also not a census but a sample. We cannot know which facility the women went to, but the assumption is she went to a nearby facility in her district. However, she could have went to facility in another district if there was no facility in her district that had fertilization procedures or was of low quality. The two data sources are three years apart however and women may have moved in this time. These methodological issues should be explained more clearly and discussed as limitations to the analysis.

Answer: We have mentioned, in the method section, that we calculated facility readiness indicators at the district level using DLHS facility data of 2012–13. We only considered the data of District Hospitals and Community Health Centers (CHC) because all DH and CHCs within the district were covered in the DLHS facility survey, and no sampling procedure is involved. We assumed that the facility readiness scores at the district level will provide a proxy estimation of the structural aspect of QoC readiness in the district.

The QoC process-outcome indicator was calculated at the individual level using NFHS 2015–16 data. For the multilevel analysis, we merged the individual-level dataset and district-level variables following ‘many to one’ procedure, so in the merged dataset all women from a district had the same score for facility readiness.

The DLHS facility survey 2012–13 does not provide data from all Indian districts and data from 560 districts are available. Therefore, we have only used the NFHS women data form only those 560 districts out of a total of 640 Indian districts in the dataset.

Yes, we do agree with the reviewer that two data sources are three years apart observations that the women could have gone to a facility in another district. While merging NFHS 2015–16 and DLHS 2012–13 datasets, we assumed that there is not much structural variation in infrastructure in the two survey periods. Due to this assumption, the changes in structural factors during this period are unaddressed. On the other hand, many of the women, who adopted sterilization, underwent the procedure well before the survey year so even if we have the data from the same year the direct one-to-one matching of facility readiness and QoC process-outcome is not possible.

In this study, DLHS 2012–13 data for ‘structure score’ serves as the proxy measurement for the system and facility readiness which is a precondition for having quality service. We considered all these issues as the limitations of the study, and we have addressed in the second last paragraph of the discussion section.

3. Other background variables that would have been important to include, are women’s number of living children, and perhaps information about the provider if available such education level or number of years of experience, or type of provider. Perhaps also if there is information on whether this was the women’s first visit to the facility. This may influence her judgment on the QoC of the facility.

Answer: The authors agree to the reviewer that those are some important variables to consider. However, the variables about a woman’s visit to the facility or the education, years of experience, or type of provider who was conducted the sterilization procedure are not available in the NFHS dataset.

In this analysis, the dependent variable is the QoC of the sterilization and the existing literature on QoC did not show that the number of children of the woman could be a determining factor for the quality of care a woman receives for sterilization procedure. Therefore, we did not consider that as an independent variable.

4. You are modeling the log odds of good to high QoC correct? In the description of your equation you said good QoC.

Answer: In revised manuscript receipt of ‘low’ QoC has been considered for modelling.

5. In the methods, you need to describe how the multilevel models were fit in Stata. To account for the multi-stage survey design, the svy command should be used and for multi-level models a weight is required for each level. Please describe which weights were used and how you accounted for the survey design. If this was not done, then you will underestimate your SEs. Was ArcGIS only used to create the maps? This was not clear.

Answer: We agree that to account for the multi-stage survey design the svy command should be used. The survey final weights are not allowed with multilevel models. In NFHS the sample weights were calculated for the cluster level (PSUs). Since the multilevel models require each stage-level weight variable using the stage’s corresponding weight such that the weights should be constant within the groups defined. In this study, the respondents comprise the first level, and the districts comprise the second level. As we didn’t consider cluster (or PSU) as a level and weights were not constant within the district, we didn’t use the sampling weights in the regression analysis. Yes, ArcGIS software was used to create the maps.

6. Since you describe low QoC in Table 1 and Figures 1 and 2, I believe it makes more sense to model low QoC in the regressions and not good to high QoC. This also makes sense since QoC is relatively high and there is little variability. Also, from a programmatic sense you would want to identify where the low QoC is for interventions.

Answer: Thank you for your comment. We have changed the coding of the dependent variable and recreated the table and text.

7. The paper is not consistent in how you refer to the outcome. Sometimes you say good QoC, sometimes excellent, sometimes very high. Please be consistent so it is not confusing.

Answer: Thank you for noting this. We have revised the manuscript addressing your suggestion.

8. Interpretation of odds ratios are not exactly correct. Example 8% less likely. These are not likelihoods. Please say 8% lower odds for instance. Also, an AOR of 0.926 rounds to 7% lower odds and not 8%. I suggest rounding the AORs to 1 or 2 decimal places and checking the numbers used in the text.

Answer: Thank you for noting this. We have revised the manuscript as per journal guideline.

9. Since most of the variables used to construct the QoC outcome are about the providers counseling or what they told the client, some literature on provider counseling or provider-client interactions would be useful both in the introduction and discussion.

Answer: Thank you for noting this. As you suggested, we have updated the manuscript.

**Reviewer #2:** This paper is very well written and the analysis has been done meticulously. It highlights an important issue of the quality of care of sterilization services in India. However, I believe that there are some places that this article could modify. First of all, there are a few corrections in the language of the article. For example, in the fourth paragraph of the discussion, in the fourth line, the word "centers" is incorrectly typed as "Canters". There are a few other grammatical mistakes for which the author should re check the article manuscript.

Next, it would be of use if the authors could specifically suggest in the article in the discussion part about what could be helpful to improve the quality of care of sterilization services at each tier of the public health system on both the provider's side and the beneficiary's side at all three structure-process-outcome levels.

Answer: We appreciate your insightful suggestions. As you suggested, we have thoroughly revised the manuscript addressing your suggestion.

6. PLOS authors have the option to publish the peer review history of their article ([what does this mean?](https://nam02.safelinks.protection.outlook.com/?url=https%3A%2F%2Fjournals.plos.org%2Fplosone%2Fs%2Feditorial-and-peer-review-process%23loc-peer-review-history&data=02%7C01%7Camozumdar%40popcouncil.org%7Cd216b84cb1ac48fab92a08d83ef1cef5%7Cda295b63c8454fa2990b1ab42d54f2ca%7C1%7C0%7C637328556557106090&sdata=OwLiYWtr8AvhOGvEIDZRbAu4zlgnongqvevBycNQSk8%3D&reserved=0)). If published, this will include your full peer review and any attached files.


If you choose “no”, your identity will remain anonymous but your review may still be made public.


**Do you want your identity to be public for this peer review?** For information about this choice, including consent withdrawal, please see our [Privacy Policy](https://nam02.safelinks.protection.outlook.com/?url=https%3A%2F%2Fwww.plos.org%2Fprivacy-policy&data=02%7C01%7Camozumdar%40popcouncil.org%7Cd216b84cb1ac48fab92a08d83ef1cef5%7Cda295b63c8454fa2990b1ab42d54f2ca%7C1%7C0%7C637328556557116082&sdata=74pc6%2BSj4%2FcvA1seS3areJnfSXtkam0Y%2BW%2FoRU09fBw%3D&reserved=0).

Reviewer #1: **Yes:** Shireen Assaf

Reviewer #2: **Yes:** Yash Alok

[NOTE: If reviewer comments were submitted as an attachment file, they will be attached to this email and accessible via the submission site. Please log into your account, locate the manuscript record, and check for the action link "View Attachments". If this link does not appear, there are no attachment files.]

While revising your submission, please upload your figure files to the Preflight Analysis and Conversion Engine (PACE) digital diagnostic tool, [https://pacev2.apexcovantage.com/](https://nam02.safelinks.protection.outlook.com/?url=https%3A%2F%2Fpacev2.apexcovantage.com%2F&data=02%7C01%7Camozumdar%40popcouncil.org%7Cd216b84cb1ac48fab92a08d83ef1cef5%7Cda295b63c8454fa2990b1ab42d54f2ca%7C1%7C0%7C637328556557116082&sdata=%2F3tLu4Tey0C0GJ%2BoQD3AaK%2FK9%2FsT5JNglzKsYYO0oOM%3D&reserved=0). PACE helps ensure that figures meet PLOS requirements. To use PACE, you must first register as a user. Registration is free. Then, login and navigate to the UPLOAD tab, where you will find detailed instructions on how to use the tool. If you encounter any issues or have any questions when using PACE, please email PLOS at [figures@plos.org](mailto:figures@plos.org). Please note that Supporting Information files do not need this step.

Answer: We have checked all our figures using PACE digital diagnostic tool before uploading.
